# A new species of the genus *Euxaldar* Fennah, 1978 (Hemiptera, Fulgoromorpha, Issidae) from China and revision on the molecular phylogeny of the family

**DOI:** 10.3897/zookeys.1021.35510

**Published:** 2021-03-01

**Authors:** Liang-Jing Yang, Zhi-Min Chang, Lin Yang, Xiang-Sheng Chen

**Affiliations:** 1 Institute of Entomology, Guizhou University, Guiyang, Guizhou, 550025, China Guizhou University Guiyang China; 2 The Provincial Special Key Laboratory for Development and Utilization of Insect Resources, Guizhou University, Guiyang, Guizhou, 550025, Chinas Liupanshui Normal College Liupanshui China; 3 College of Animal Science, Guizhou University, Guiyang, Guizhou, 550025, China Guizhou University Guiyang China; 4 Office of Academic Affairs, Liupanshui Normal College, Liupanshui, Guizhou, 55300, China Liupanshui Normal College Liupanshui China

**Keywords:** Checklist, DNA sequence, Hemisphaeriini, identification key, morphology, planthopper, taxonomy

## Abstract

A new species *Euxaldar daweishanensis* Yang, Chang & Chen, **sp. nov.** is described and illustrated from southwestern China. The female genitalia of the genus *Euxaldar* is described and presented for the first time. A checklist and key to the known species of the genus are provided. A revised molecular phylogenetic analysis of the family Issidae based on combined partial sequences of *18S*, *28S*, *COI*, and *Cytb* is provided using both Maximum likelihood and Bayesian inference analyses.

## Introduction

The planthopper genus *Euxaldar* Fennah, 1978 is a small group in the Issidae tribe Hemisphaeriini Melichar, 1906, established for a single species *E. jehucal* Fennah, 1978, recorded from Ninh Binh, Ha Noi, Vinh Phuc, Hoa Binh, and Haiphong Province in northern Vietnam (Fennah 1978; Gnezdilov and Constant 2012). Recently Gnezdilov et al. (2017a) reviewed the genus and described *E. lenis* Gnezdilov, Bourgoin & Wang, 2017 from Lam Dong Province (Da Lat) of southern Vietnam. Later, Zhang et al. (2018) recorded the genus for the first time from southeastern China and described *E. guangxiensis* Zhang, Chang & Chen, 2018 from Guangxi Province. Previously, Gnezdilov placed *Euxaldar* into the tribe Issini Spinola, 1839 (Gnezdilov 2013). However, Wang et al. (2016) moved it to HemisphaeriiniMongolianina. Recently, this genus was placed in subgroups of Mongolianina: *Mongoliana* + (*Euxaldar* + *Macrodaruma*) by Zhao et al. (2019), but as shown in this study it is better placed in the subgroup (*Retaldar* + (*Clypeosmilus* + Eusudasina)) because all genera in this subgroup have the same characteristic protruded clypeus.

Below, we describe and illustrate a new species of *Euxaldar* from Yunnan Province in China, provide a checklist and key to *Euxaldar* species, and describe and photograph the female genitalia of the new species. The partial DNA sequences (*16S*, *28S* (d6-d7), *COI*, *Cytb*) of the new species are briefly analyzed. A revised molecular phylogeny is analyzed by Bayesian and Maximum likelihood based on seven sequences of four genes (*18S*, *28S*, *COI* and *Cytb*), providing molecular evidence of phylogenetic relationships within the Issidae and enabling a revaluation of the current classification of the family Issidae by Wang et al. (2016), Zhao et al. (2019) and Gnezdilov et al. (2020).

## Materials and methods

The morphological terminology used for body appearance follows Chan and Yang (1994) and Anufriev and Emeljanov (1988). Forewing venation pattern follows Bourgoin et al. (2015). The terminologies of male and female genitalia follow Bourgoin (1987, 1993) and Chang et al. (2015). Body length (included forewings) is given in millimeters (mm).

The genital segments of the specimens were macerated in a boiling solution of 10% NaOH for about 5 minutes, washed in distilled water, then immersed in glycerine for observation, dissection, drawing, and photography. They were stored in a micro vial in glycerol for further examination. A Leica MZ 12.5 stereomicroscope was used for illustrations. A KEYENCE VHX-1000C was used to acquire photographs. All specimens studied are deposited in the Institute of Entomology, Guizhou University, Guiyang, China (**GUGC**).

The molecular phylogenetic study included 71 species belonging to 48 genera as ingroups from Issidae (Wang et al. 2016; Zhao et al. 2019; Gnezdilov et al. 2020) and five species as outgroups from the families Cixidae, Caliscelidae, Delphacidae, Dictyopharidae, Tropiduchidae. Data for the 71 included species were downloaded from NCBI. Five ingroup species including the new species were newly sequenced, for which total DNA was extracted using the Animal Tissue Genomic DNA Kit (Tiangen Biotech Company, Beijing, China). Primers and PCR procedures are listed in Tables 1, 2 and carried out in 30 μl volume reaction. Accession numbers for species used in the phylogenetic analysis are shown in Table 4.

The DNA sequencing was performed at Sangon Company (Shanghai, China). Sequence chromatograms were checked and assembled by Seqman from the package DNAstar v5.01 (www.dnastar.com), calculated by MEGA 6.06 and Notepad 7.6.2. The Maximum likelihood (ML) phylogenetic analysis was performed by IQtree v1.6.7 and visualized by Figtree v1.1.2. A Bayesian estimation search (BI) was performed using MrBayes (Ronquist et al. 2011) on the CIPRES Science Gateway V3.1 Portal (https://www.phylo.org/portal2/home.action). Best partitions and models were chosen by PartitionFinder 2 (Lanfear et al. 2017), running conditions as described in Appendix 1.

**Table 1.** Primers used for amplification and sequencing.

Gene	Primer	Sequence (5^’^–3^’^)
*COI*	COI (LCO18)-PF	GGTCAACAAATCATAAAGATATTG
	COI (HCO29)-PR	TAAACTTCAGGGTGACCAAAAAAT
*16S* (Clary and Wolstenholme 1985)	16S-PF	GCCTGTTTATCAAAAACAT
	16S-PR	CCGGTCTGAACTCAGATCA
*Cytb* (Bourgoin et al. 1997)	Cytb-PF	TATGTACTACCATGAGGACAAATATC
	Cytb-PR	ATCTTAATGCAATAACTCCTCC
*28S* d6–d7 (Cryan et al. 2000)	28S EE	CCGCTAAGGAGTGTGTAA
	28S MM	GAAGTTAGGGATCTARTTTG
*28S* d3–d5 (Belshaw and Quicke 2002)	28S Ai	GACCCGTCTTGAAACACG
	28S D4D5r	GTTACACACTCCTTAGCGGA

**Table 2.** PCR procedures.

Gene	*COI*	*16S*	*Cytb*	*28S* d3-d5	*28S* d6-d7
Initial denaturation	94 °C 5 min	95 °C 7 min	94 °C 5 min	94 °C 3 min	94 °C 3 min
95 °C 7 min	94 °C 30 sec	95 °C 50 sec	94 °C 1 min	94 °C 1 min	94 °C 1 min
Annealing	55 °C 1 min	50 °C 1 min	47 °C 1 min	54 °C 1 min	55 °C 1 min
Extension	72 °C 1 min	72 °C 1 min	72 °C 1 min	72 °C 1 min	72 °C 1 min
Cycles	35 Cycles	35 Cycles	35 Cycles	35 Cycles	40 Cycles
Annealing	72 °C 10 min	72 °C 10 min	72 °C 10 min	72 °C 10 min	72 °C 10 min

## Taxonomy

### Genus Euxaldar

Taxon classificationAnimaliaHemipteraIssidae

Fennah, 1978

AD5DDF68-FD7C-509C-BBC3-0F0733FB86B8

Euxaldar Fennah, 1978: 267.

#### Type species.

*Euxaldar jehucal* Fennah, 1978, by monotypy.

#### Diagnosis.

Coryphe transverse, 2–3 times as wide as long. Metope flat and elongate, disc smooth or densely covered by pustules. Anteclypeus with distinct median carinae. Forewings with costal margin basally angled and convex below eyes, claval suture developed, venation hazily reticulate, CuP distinct. Hind tibia with 2 lateral spines. Spinal formula of hind leg (7–9)–(6–8)–2. Pygofer with posterior margin distinctly convex. Male anal tube apically enlarged or elongated in dorsal view. Periandrium asymmetrical.

#### Distribution.

China, Vietnam.

### Checklist of *Euxaldar* species

*E. daweishanensis* sp. nov. (Southwestern China: Yunnan Province)

*E. guangxiensis* Zhang, Chang & Chen, 2018 (Southeastern China: Guangxi Province)

*E. jehucal* Fennah, 1978 (Northern Vietnam: Ninh Binh, Ha Noi, Vinh Phuc, Hoa Binh, and Haiphong Provinces)

*E. lenis* Gnezdilov, Bourgoin & Wang, 2017 (Southern Vietnam: Lam Dong Province)

### Key to male species of *Euxaldar*

Modified from Gnezdilov et al. (2017a) and Zhang et al. (2018).

**Table:** 

1	Metope smooth. Forewings without coloured bands or spots (Gnezdilov et al. 2017a: fig. 23)	***E. lenis***
–	Metope with a row of distinct pustules along lateral margins. Forewings with coloured bands or spots (Figs 9, 11; Zhang et al. 2018: fig. 5; Gnezdilov et al. 2017a: figs 20, 33)	**2**
2	Metope without median carinae. Metopoclypeal suture incomplete medially. Hind wings rudimentary, shorter than half length of forewings (Zhang et al. 2018: fig. 5)	***E. guangxiensis***
–	Metope with weak median carinae running from upper margin to middle. Metopoclypeal suture complete, straightly, or weakly concave. Hind wings developed, longer than half length of forewings (Gnezdilov et al. 2017a: figs 20, 33)	**3**
3	Coryphe about 3 times as wide as long in the middle. Male anal tube enlarging from base to apical margin and deeply concave at posteromedial part in dorsal view (Gnezdilov et al. 2017a: fig. 6)	***E. jehucal***
–	Coryphe about 4 times as wide as long in middle. Male anal tube elongated in dorsal view, enlarging from base to apical fourth and narrowing at apical part, lateral margins with a triangular process in the upper half on each side (Figs 8, 13)	***E. daweishanensis* sp. nov.**

### Euxaldar daweishanensis sp. nov.

Taxon classificationAnimaliaHemipteraIssidae

86CF985D-341E-5374-B39F-3A16DCD5DB30

http://zoobank.org/663A901A-6FF8-4BC9-A6B9-C9D1244AAB5B

Figs 1–7 , 8–17 , 19–26

#### Type material.

***Holotype***: ♂, **China**: Yunnan Province, Pingbian County, Mt: Daweishan National Nature Reserve (23°07'N, 103°20'E), 8 August, 2017, Qiang Luo, Nian Gong, Y.-J Sui, Yan Zhi. ***Paratypes***: 7♂♂ 36♀♀, same data as holotype.

#### Measurements.

Total length (from apex of coryphe to tip of forewing): male 4.1–4.3 mm (*N* = 6), female 4.6–4.9 mm (*N* = 10); forewing length: male 3.8–4.0 mm (*N* = 7), female 4.2–4.4 mm (*N*= 10).

#### Diagnosis.

This species differs from other *Euxaldar* species by the following characters: (1) coryphe about 2.3 times wider than long (less, or more than 2.3 times as wide as long in other species of *Euxaldar*); (2) first metatibiotarsal of hind leg with 8 intermediate spines (other species of *Euxaldar* with first metatarsomere of hind leg with 6 or 7 intermediate spines); (2) penis with 3 different ribbon-shaped processes at middle (Figs 16, 17, pp, paed), dorsal lobe of periandrium with 2 asymmetrical sword-shaped subapical processes in apical half (Figs 16, 17, sap) (other species without sword-shaped subapical processes in apical half of dorsal lobe of periandrium).

**Figures 1–7.**
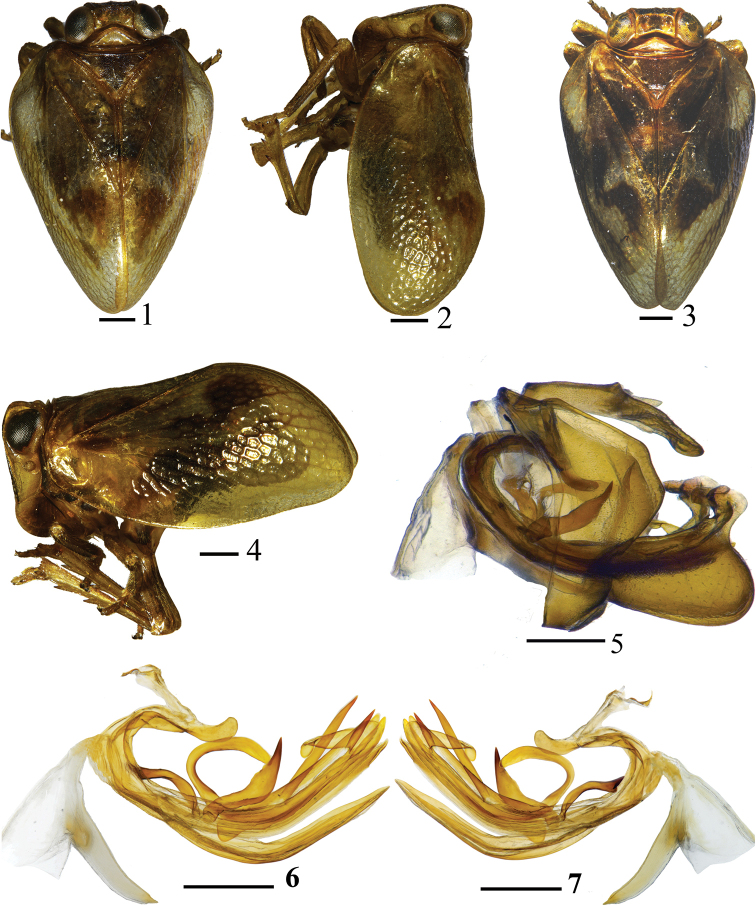
*Euxaldar daweishanensis* sp. nov. (adult) **1** male dorsal view **2** male lateral view **3** female dorsal view **4** female lateral view **5** male genitalia, lateral view **6** penis, left lateral view **7** penis, right lateral view. Scale bars: 0.5 mm.

#### Coloration.

**Figures 8–18.**
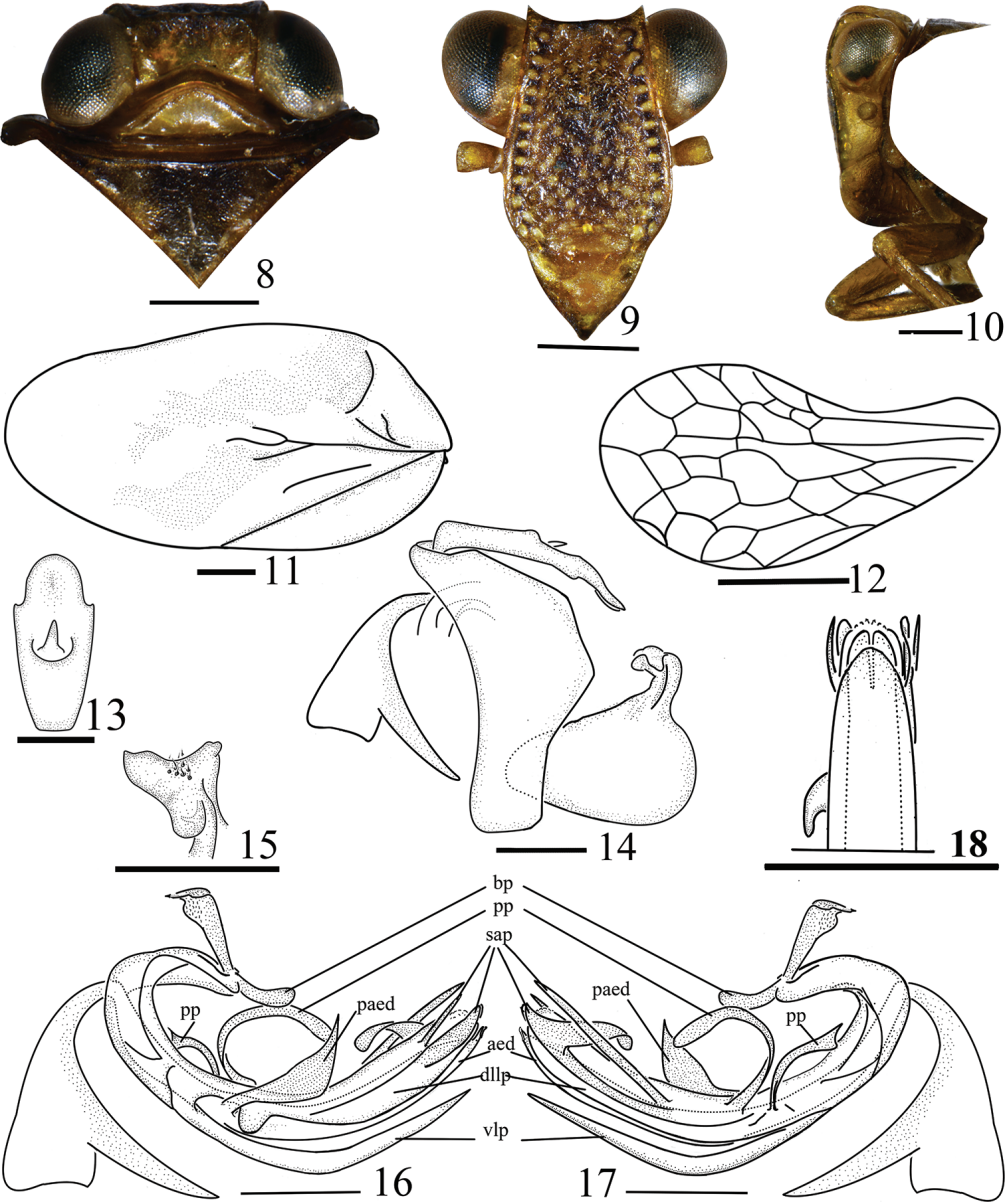
*Euxaldar daweishanensis* sp. nov. (male adult) **8** head and thorax, dorsal view **9** face, front view **10** head and thorax, lateral view **11** forewings **12** hind wing **13** anal tube, dorsal view **14** pygofer, anal tube and genital style, lateral view **15** capitulum of gonostyli, dorsal view **16** penis, lateral view (left) **17** penis, lateral view (right) **18** penis, ventro-apical view. Abbreviations: **aed**–aedeagus; **bp**–basal process of the periandrium; **dllp**–dorso-lateral lobe of periandrium; **paed**–process of aedeagus; **pp**–process of periandrium; **sap**–subapical processes of periandrium; **vlp**–venteral lobe of periandrium. Scale bars: 0.5 mm

Male body brown yellowish, with irregular dark brown bands on forewings. Coryphe brown (Fig. 8). Metope with all margins, pustules, and median carinae pale yellow, disc dark brown (Fig. 9). Metopoclypeal suture light yellow. Anteclypeus straw yellow. Postclypeus pale yellow (Figs 9, 10). Rostrum and antenna straw yellow (Fig. 10). Eyes dark brown (Figs 8–10). Pronotum straw yellow. Mesonotum dark brown (Fig. 8). Forewings slightly hyaline, with 2 irregular brown bands (Figs 1, 2, 11): a large one derived from costal margin to almost C2 of radial cell, small one derived from apical half of median cell, extended to areola postica (anterior cubital area). Legs (Figs 2, 4) light brown. Abdomen brown, male genital segment light straw yellow. Females generally darker than males (Figs 3, 4).

#### Head and thorax.

Coryphe transverse, about 2.3 times wider than long, anterior margin weakly prominent in the middle, posterior margin angularly concave (Fig. 8). Metope flat, median carinae weak, running from upper margin and reaching middle, with a row of distinct pustules along lateral margins, disc with weak pustules (Fig. 9). Metopoclypeal suture complete (Fig. 9). Anteclypeus with distinct median carinae (Figs 9, 10). Pronotum with disc depressed (Fig. 8). Mesonotum about 2.1 times longer than pronotum. Forewings (Figs 1–4, 11) with distinct claval suture and CuP venation, the other venation reticulate, poorly recognizable. Hind wings about 0.7 times as long as forewings, venation reticulate (Fig. 12). Hind tibiae with 2 lateral teeth. Metatibiotarsal formula (9–8)–8–2.

#### Male genitalia.

Anal tube (Fig. 13) enlarging from base to apical fourth in dorsal view, narrowing to apex, apical margin convex in the middle, laterally with 2 small triangular processes in apical fourth. Pygofer with hind margin distinctly convex (Figs 5, 14). Gonostyli triangular, hind margin convex, caudo-dorsal angle rounded (Fig. 14). Capitulum of gonostyli style with wide and short neck, with a wide lateral tooth and 2 apical teeth (Figs 14, 15). Corpus of connective rod-like (Figs 5–7, 16, 17), curved, cuticularized, reaching middle of periandrium; tectiductus of connective cup-shaped, third ventral part separated from corpus (Fig. 14). Periandrium asymmetrical (Figs 6, 7, 16, 17), suspensorium V-shaped in dorsal view, membranaceous in the middle; base with process claval (Figs 16, 17, bp), dorsal periandrium lobe with 2 ribbon-like processes in center near right edge (Figs 16, 17, pp), directed dorsad, respectively curved caudad and cephalad; dorsal lobe in left lateral view with 2 subapical processes near apex (Fig. 16, sap): one crescent-shaped, above base with another process shortly sword-shaped, directed caudad; in right lateral view (Fig. 17, sap) with two subapical processes derived from apical third, directed apically, one process base movable, sword-shaped, below base another process crutch-like and sclerotized. Ventral periandrium lobe (Fig. 18, vlp) with apical margin convex, shorter than dorso-lateral lobe of periandrium (Figs 16, 17, dllp, 18) in ventral view. Aedeagus (Figs 16, 17, aed) with dagger-shaped process, base slightly movable, directed dorsad, slightly inclined caudad (Figs 16, 17, paed).

**Figures 19–26.**
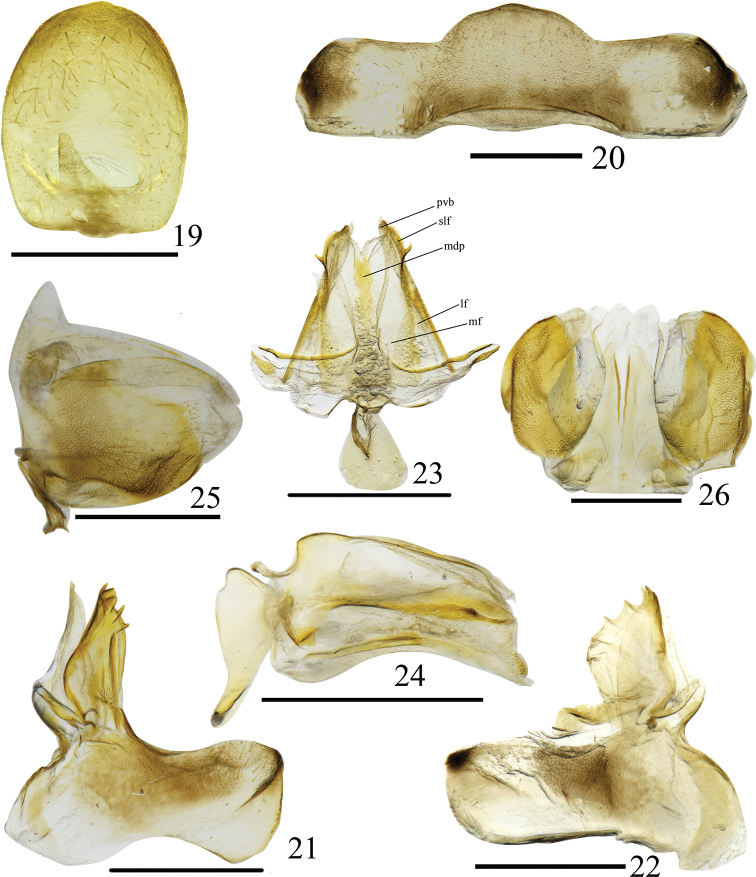
*Euxaldar daweishanensis* sp. nov. (female adult) **19** female anal tube, dorsal view **20** sternite VII, ventral view **21, 22** gonocoxa VIII and gonapophysis VIII, ventral view **23** gonapophysis IX and gonaspiculum bridge, dorsal view **24** gonapophysis IX and gonaspiculum bridge, lateral view **25** gonoplacs, lateral view **26** gonoplacs, dorsal view Abbreviations: **lf**–lateral field of posterior connective lamina of gonapophyses IX; **mdp**–medial dorsal process; **mf**–medial field of posterior connective lamina of gonapophyses IX; **pvd**–posterior ventral lobes; **slf**–sublateral field of posterior connective lamina of gonapophyses IX. Scale bars: 0.5 mm.

#### Female genitalia.

Anal tube ovate in dorsal view, about 1.3 times longer than maximal width at second part (Fig. 19). Anal style long, located at basal fifth of anal tube. In ventral view, Sternite VII with hind margin convex medially, without any process in ventral view, disc arched ventrad (Fig. 20). Anterior connective lamina of gonapophysis VIII nearly rectangular, with 3 or 4 apical teeth on inner lateral margin and 3 lateral teeth bearing 3 keels on outer lateral margin (Figs 21, 22); endogonocoxal lobe developed, membranous in distal part (Figs 21, 22). Posterior connective lamina of gonapophyses IX triangular in dorsal view (Fig. 23), narrowing; median field with leaf-like process bearing apical margin, deeply incised in the middle (Fig. 23, mdp); lateral field (Fig. 23, lf) without obvious process; distal parts of laminae (Fig. 23, slf) with tooth-like process on each lateral margin; posterior ventral lobes bent at slender angle (Figs 23, pvb, 24). Gonoplacs in lateral view irregularly elliptical (Fig. 25), without carinae, with apical half fused, apical margin membranous (Fig. 26).

#### Etymology.

This new species is named after the type locality, Mt. Daweishan National Nature Reserve, Yunnan Province, China.

#### Distribution.

China (Yunnan Province)

#### Remark.

This new species resembles *Euxaldar jehucal* but differs from the latter by the following combined features: Anal tube with apical margin convex in the middle, lateral margin with a small triangular process in each side (anal tube wide, apical margin deeply concave medially in *E. jehucal*); periandrium with two asymmetrical subapical processes sword-shaped in apical half (periandrium with subapical processes not as sword-shaped in *E. jehucal*); aedeagus with one medial dagger-like process on lateral margins (aedeagus without any processes on lateral margins in *E. jehucal*).

#### Phylogenetical analysis.

Four gene fragments of *Euxaldar daweishanensis* sp. nov. were sequenced and registered in GenBank with the accession numbers as follows: MK441660 (*COI*), MK426664 (*16S*), MK441661 (*Cytb*), MK441662 (*28S* d6-d7). Nucleotide compositions are listed in Table 3. A+T content of *16S* is the highest (76.0%) and *28S* (d6-d7) is the lowest (39.2%).

**Figure 27.**
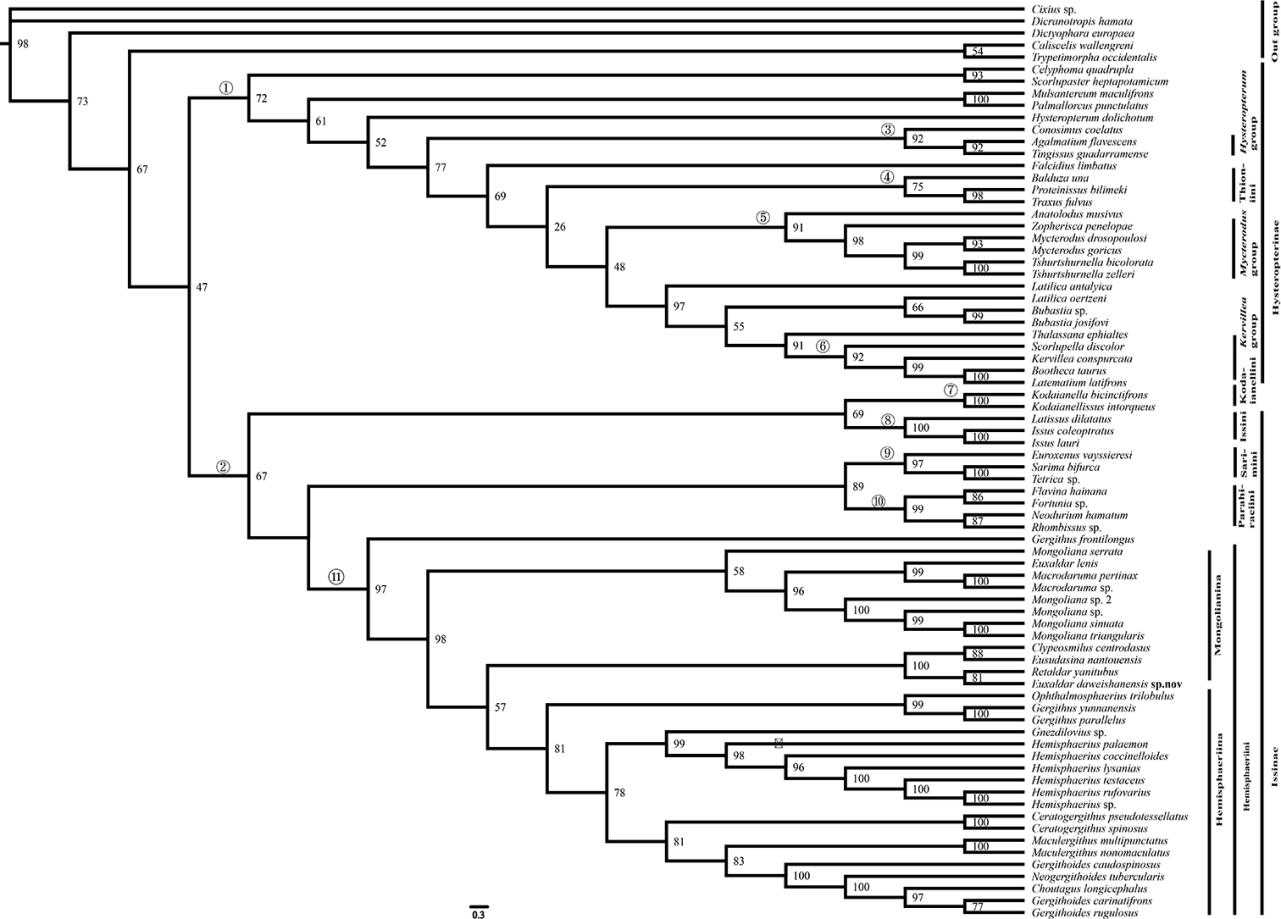
Maximum likelihood (ML) tree estimated from the combined based on combined sequences (*18S*, *28S*, *COI*, *Cytb*). At each node, values indicate bootstrap supports.

**Figure 28.**
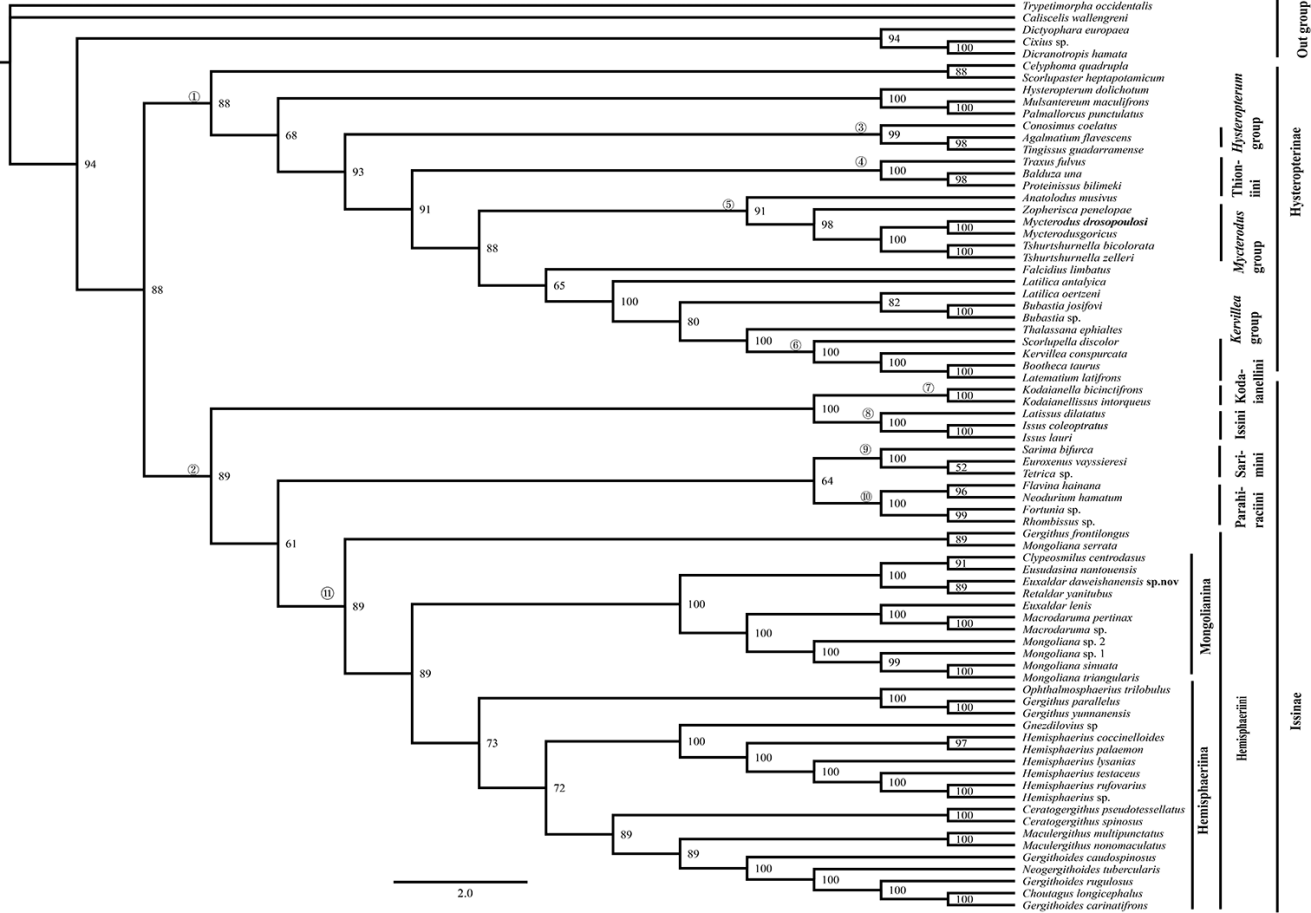
Bayesian 50% consensus tree based on combined dataset. Nodes of the major clades are numbered and refer to text. Each node is documented with its posterior probability (PP) value.

This study deals with more molecular markers from Oriental and Western Palaearctic, Nearctic and Neotropical regions than previous reviews by Wang et al. (2016) and Gnezdilov et al. (2020). BI (Fig. 27) and ML (Fig. 28) topologies were mostly congruent, and the monophyly of Issidae was reconfirmed. The Issidae had lower support in the ML tree (BS: 47) than Gnezdilov et al. (2020) and Wang et al. et al. (2016) and higher support in the BL analysis (PP: 88). Subfamilies Hysteropterinae Melichar, 1906 sensu (Gnezdilov et al. 2020) and Issinae Spinola, 1839 sensu (Gnezdilov et al. 2020) are both recovered (nodes 1 and 2: ML: 47, 67; BI: 88, 89, respectively).

**Table 3.** Nucleotide gene composition of *Euxaldar daweishanensis* Yang, Chang & Chen, sp. nov.

Gene	A%	T%	G%	C%	A+T%
*COI*	33.2	36.3	17.8	12.7	69.5
*16S*	27.4	48.6	14.9	9.1	76.0
*Cytb*	35.3	34.5	11.6	18.6	69.8
*28S* d6-d7	20.4	18.9	33.7	27.0	39.2

Node 1 includes almost all tribal level genera group of the subfamily Hysteropterinae sensu Gnezdilov (2016a, b, 2020) and the tribe Thioniini Melichar, 1906 sensu (Gnezdilov 2018): 1) Node 4 (ML: 75, BI: 100) corresponds to the subtribe Thioniina sensu Gnezdilov (2018) with the inclusion of American taxa, characterized by hind wings reduced or rudimentary, A2 vein branched; 2) Nodes 5 and 6 corresponds to the monophyletic *Kervillea*, and *Mycterodus* genera group sensu Gnezdilov (2016 a, b); monophyly of the *Hysteropterum* genera group was not supported by this analysis (node 3).

Node 2 (ML: 67, BI: 89) includes five monophyletic tribes (nodes 7–11): Issini, Kodaianellini and Hemisphaeriini sensu (Gnezdilov 2020), Parahiraciini, and Sarimini sensu (Wang et al. 2016), while the monophyly of Sarimini and Parahiraciini was not supported by Gnezdilov (2020).

**Table 4.** Species used in the phylogeny analysis with accession number. “*” denotes new added sequences in this study.

Species	*COI*	*Cytb*	Gene *18S* (*A2–9R*)	Gene *28S* (*D3–D5*)	Gene *28S* (*D6–D7*)	Collection
*Agalmatium flavescens* (Olivier, 1791)	MN194180	MN191521	MN165781	MN266987	MN266956	Russia
*Anatolodus musivus* Dlabola, 1982	MN194181	–	MN165782	MN266988	MN266957	Turkey
*Balduza una* (Ball, 1910)	–	MN191522	MN165783	MN266989	MN266958	Mexico
*Bootheca taurus* (Oshanin, 1870)	MN194182	MN191523	MN165784	MN266990	MN266959	Bulgaria
*Bubastia josifovi* Dlabola, 1980	–	MN191524	MN165785	MN266991	MN266960	Bulgaria
*Bubastia* sp.	–	MN191525	MN165786	MN266992	MN266961	Greece
*Caloscelis wallengreni* Stål, 1863	KX702956	KX702901	KX702855	KX761436	KX702877	China
*Celyphoma quadrupla* Meng & Wang, 2012	KX702919	KX702906	KX761576	KX761444	KX702806	China
*Ceratogergithus pseudotessellatus* (Che, Zhang & Wang, 2007)	KX761502	KX761513	KX761491	KX761532	KX761521	China
*Ceratogergithus spinosus* (Che, Zhang & Wang, 2007)	KX761502	KX761513	KX761491	KX761532	KX761521	China
*Choutagus longicephalus* Zhang, Wang & Che, 2006	KX761460	–	KX650620	KX761450	KX702810	China
*Cixius* sp.	KR343731	KX702891	JQ982514	KX761413		France
*Clypeosmilus centrodasus* Gnezdilov & Soulier-Perkins, 2017	KX761470	KX761474	KX761575	–	–	Vietnam
*Conosimus coelatus* Mulsant & Rey, 1855	MN194183	MN191526	MN165787	MN266993	MN266962	France
*Dicranotropis hamata* (Boheman, 1847)	KX76146	–	KX702837	KX761409	–	Austria
*Dictyophara europaea* (Linnaeus, 1767)	KJ911190	KX702896	KX702851	KX761427	–	Russia
*Euroxenus vayssieresi* (Bonfils, Attie & Reynaud, 2001)	–	–	MN165789	MN266995	MN266964	China, Reunion
*Eusudasina nantouensis* Yang, 1994	HM052838	HM452266	–	–	–	China
*Euxaldar daweishanensis* sp. nov.*	MK441660	MK441661	–	–	MK441662	China
*Euxaldar lenis* Gnezdilov, Bourgoin & Wang, 2017	–	–	KX761565	KX761412	–	Vietnam
*Falcidius limbatus* (A. Costa, 1864)	MN194185	–	MN165790	MN266996	MN266965	Italy
*Flavina hainana* (Wang & Wang, 1999)	–	KX702912	KX702824	KX761453	MN381846	China
*Fortunia* sp.	KX761498	KX761509	KX761487		KX761518	China
*Gergithoides carinatifrons* Schumacher, 1915	KX761555	KX702905	KX761538	–	KX702805	China
*Gergithoides caudospinosus* Chen, Zhang & Chang, 2014*	MN171521	MW233581	–		MW228374	China
*Gergithoides rugulosus* (Melichar, 1906)	HM052835	HM452279	–	–	–	China
*Gergithus frontilongus* Meng, Webb & Wang, 2017*	MN171522	MW233582	–		MW228375	China
*Gergithus parallelus* Che, Zhang & Wang, 2007*	MN171523	MW233583	–		MW228376	China
*Gergithus yunnanensis* Che, Zhang & Wang, 2007	KX702924	KX702915	KX702831	KX761456	MN381848	China
*Gnezdilovius* sp.*	MN171524	–	–	–	MW228377	China
*Hemisphaerius coccinelloides* (Burmeister, 1834)	KX702934	KX702884	KX702834	KX761405	KX702861	Philippines
*Hemisphaerius lysanias* Fennah, 1978	KX702933	KX702883	KX702833	KX761404	KX702860	Vietnam
*Hemisphaerius palaemon* Fennah, 1978	KX761497	KX761508	KX761486	KX761526	KX761517	China
*Hemisphaerius rufovarius* Walker, 1858	KX702923	KX702913	KX702825	KX761454	KX702812	China
*Hemisphaerius* sp.	KX761556	KX702885	KX702835	KX761406	KX702862	Laos
*Hemisphaerius testaceus* Distant, 1906	HM052831	HM452258	–	–	–	China
*Hysteropterum dolichotum* Gnezdilov & Mazzoni, 2004	–	–	MN165791	MN266997	MN266966	France
*Issus coleoptratus* (Fabricius, 1781)	KX702932	KX761550	KX761568	KX761403	KX761560	France
*Issus lauri* Ahrens, 1814	–	MN191528	MN165793	MN266999	MN266968	Italy
*Kervillea conspurcata* (Spinola, 1839)	MN194187	MN191529	MN165794	MN267000	MN266969	Slovenia
*Kodaianella bicinctifrons* Fennah, 1956	KX761458	KX702902	KX702814	KX761441	KX702802	China
*Kodaianellissus intorqueus* Wang, Bourgoin & Zhang, 2017	–	KX761472	KX761476	KX761480	KX761482	China
*Latematium latifrons* (Fieber, 1877)	MN194188	MN191530	MN165795	MN267001	MN266970	Bulgaria
*Latilica antalyica* (Dlabola, 1986)	–	MN191531	MN165796	MN267002	MN266971	Greece
*Latissus dilatatus* (Fourcroy, 1785)	–	MN191532	MN165797	MN267003	MN266972	Greece
*Macrodaruma pertinax* Fennah, 1978	KX702931	KX702882	KX702832	KX761402	KX702859	Vietnam
*Macrodaruma* sp.	KX702927	KX702881	KX702828	KX761399	KX702857	China
*Maculergithus multipunctatus* (Che, Zhang & Wang, 2007)	KX702918	KX702904	KX702816	KX761443	KX702804	China
*Maculergithus nonomaculatus* (Meng & Wang, 2012)	KX761503	KX761514	KX761492	KX761533	KX761522	China
*Mongoliana serrata* Che, Wang & Chou, 2003	HM052830	HM452272	–	–	–	China
*Mongoliana sinuata* Che, Wang & Chou, 2003	KX761459	KX702908	KX702820	KX761448	–	China
*Mongoliana* sp. 2	–	–	KX761566	KX761534	MN381849	China
*Mongoliana* sp.1	–	MN332233	MN422135	MN381854	–	Thailand
*Mongoliana triangularis* Che, Wang & Chou, 2003	–	KX761510	KX761561	KX761528	–	China
*Mulsantereum maculifrons* (Mulsant & Rey, 1855)	KX702928	KX761551	KX761569	KX761400	MN381847	France
*Mycterodus drosopoulosi* Dlabola, 1982	MN194189	MN191533	MN165798	MN267004	MN266973	Greece
*Mycterodus goricus* (Dlabola, 1958)	MN194190	MN191534	MN165799	MN267005	MN266974	Greece
*Neodurium hamatum* Wang & Wang, 2011	KX702920	–	KX702818	KX761446	MN381844	China
*Neogergithoides tubercularis* Sun, Meng & Wang, 2012	KX761558	KX702910	KX702822	KX761451	MN381845	China
*Ophthalmosphaerius trilobulus* (Che, Zhang & Wang, 2006)	KX761462	KX702914	KX702826	KX761455	KX702813	China
*Palmallorcus punctulatus* (Rambur, 1840)	KX761462	KX702914	MN165800	MN267006	MN266975	Greece
*Proteinissus bilimeki* Fowler, 1904	MN194193	MN191537	MN165803	MN267009	MN266978	Greece
*Retaldar yanitubus* sp. nov.	MN381857	MN332232	MN381856	MN381853	MN381851	China
*Rhombissus* sp.		MN332231	MN381855	MN381852	MN381850	China
*Sarima bifurca* Meng & Wang, 2016	KX702921	KX761552	KX702819	KX761447	KX702808	China
*Scorlupaster heptapotamicum* Mitjaev, 1971	–	–	–	MN267010	MN266979	Kazakhstan
*Scorlupella discolor* (Germar, 1821)	–	–	MN165804	MN267011	MN266980	Bulgaria
*Tetrica* sp.	KX702922	KX702909	KX702821	KX761449	KX702809	China
*Thalassana ephialtes* (Linnavuori, 1971)	MN194194	MN191538	MN165805	MN267012	MN266981	Turkey
*Tingissus guadarramense* (Melichar, 1906)	KX702935	KX702886	MN165806	MN267013	MN266982	Portugal
*Traxus fulvus* Metcalf, 1923	MN194195	MN191539	MN165807	MN267014	MN266983	Mexico
*Trypetimorpha occidentalis* Huang & Bourgoin, 1993	KX702957	–	KX761546	KX761437	–	Kazakhstan
*Tshurtshurnella bicolorata* Gnezdilov & Oezgen, 2018	MN194196	MN191540	MN165808	MN267015	MN266984	Turkey
*Tshurtshurnella zelleri* (Kirschbaum, 1868)	–	MN191541	MN165809	MN267016	MN266985	Italy
*Zopherisca penelopae* (Dlabola, 1974)	–	–	MN165810	MN267017	MN266986	Greece

## Discussion

According to our analysis, the tribe Thioniini was recovered as monophyletic, split from the subfamily Issinae sensu Gnezdilov et al. (2020), and placed in the subfamily Hysteropterinae sensu Gnezdilov et al. (2020). Herein, we suggest that the subtribe Thioniina sensu Gnezdilov et al. (2020) should be a tribe of Hysteropterinae (Thioniini + tribal level groups of genera (Gnezdilov 2016a, b)), sharing the common characteristic of this subfamily: hind wings reduced or rudimentary. Conversely, most taxa of the subfamily Issinae Spinola, 1839 sensu Gnezdilov et al. (2020) have developed hind wings. The Issinae, including five tribes distributed in the Oriental Region, is recovered and well supported in the following topology (node 2): [(Kodaianellini, Issini) + (Sarimini, Parahiraciini) + Hemisphaeriini)].

The monophyletic tribe Hemisphaeriini Melichar, 1906 is confirmed by our data, characterized by hemispherical forewings and single-lobed or rudimentary hind wings (Gnezdilov et al. 2020). The monophyly of the subtribes Hemisphaeriina and MongolianinaWang et al. (2016) is not supported: the genus *Gergithus* shows a sister relationship with (Hemisphaeriina + Mongolianina) in this analysis (ML: 97, BI: 89).

*Mongoliana serrata* Che, Wang & Chou, 2003 is isolated from *Mongoliana* Distant, 1909 (ML:58, BI:89), confirming the hypothesis of Meng et al. (2017) that the genus *Mongoliana* could be divided into two species groups. It probably contains two different genera: one of them *M. serrata* is a new genus with the smooth frons, pale brown tegmina having dark fasciae and spots and the ventral hooks of the aedeagus variable in shape and usually unparallel. *Gergithus yunnanensis* and *G. parallelus* show a sister relationship with *Ophthalmosphaerius* Gnezdilov, 2017, probably belonging to a new genus with *Ophthalmosphaerius*; this finding agrees with Gnezdilov (2017c) and Zhao et al. (2019), but we still consider it incertae sedis until more evidence is presented.

The third lineage of Mongolianina (Zhao et al. 2019) is recovered only in our ML analysis. *Euxaldar daweishanensis* sp. nov. and *E. lenis* are grouped into a different cluster in our research: the genera of the cluster ((*E. daweishanensis* sp. nov. + *Retaldar*) + (*Clypeosmilus* + *Eusudasina*)) share a protruded clypeus, and forewings CuP clear; another cluster (*E. lenis + Macrodaruma*) recovered by Zhao et al. (2019) share a smooth metope without pustules, and sexual dimorphism. *Euxaldar lenis* probably belongs to a new genus.

*Euxaldar* is similar to the genus *Paramongoliana* Chen, Zhang & Chang, 2014 which is here formally placed in the subtribe Mongolianina according to Wang et al. (2016), but differs by the following characters: metope smooth or with pustules (metope roughly corrugated, without pustules in *Paramongoliana*, see Chen et al. 2014: figs 2–33E); forewings with CuP distinct (forewings with CuP poorly recognizable in *Paramongoliana*, see Chen et al. 2014: figs 2–33A, B, F); anal tube with apical margin not straight (anal tube nearly quadrilateral, apical margin straight in *Paramongoliana*, see Chen et al. 2014: figs 2–33H).

The genus *Euxaldar* is also similar to the genus *Clypeosmilus* (Gnezdilov et al. 2017b) in having forewings with reticulate venation and a distinct claval suture, but can differ from the latter in the following characters: postclypeus with complete median carina and anteclypeus with distinct median carina (*Clypeosmilus* with postclypeus large, flattened laterally, bearing a thick chisel-like median carina); periandrium asymmetrical (periandrium symmetrical, with pair of long and narrow subapical processes directed apically).

*Euxaldar daweishanensis* sp. nov., *E. jehucal*, and *E. guangxiensis* share several compelling characters: 1) *E. daweishanensis* sp. nov., *E. jehucal*, and *E. guangxiensis* share a metope disc with relatively weak pustules distributed in a row along the lateral margins; and 2) *E. daweishanensis* sp. nov. and *E. guangxiensis* have an anal tube with a triangular process on each lateral margin (Fig. 13; Zhang et al. 2018: figs 12, 13). Other noteworthy characters: 1) *E. guangxiensis* exhibits a vestigial hind wing; 2) *E. lenis* has a smooth metope without pustules, and sexual dimorphism. All species of this genus probably belong to different species groups or even different genera. More molecular data and other convincing morphological evidence are expected in the future, enabling further discussion of the taxonomic status of *Euxaldar*.

## Supplementary Material

XML Treatment for Euxaldar

XML Treatment for Euxaldar daweishanensis

## Appendix 1

Partitions and models used for the Maximum likelihood tree in IQtree and Bayesian 50% consensus tree.

#nexus

begin sets;

charset Subset1 = 1–1899;

charset Subset2 = 1900–2617;

charset Subset3 = 2618–3473;

charset Subset4 = 3474–4194;

charset Subset5 = 4195–4861;

charpartition PartitionFinder = GTR+I+G: Subset1, GTR+I+G: Subset2, GTR+I+G: Subset3, GTR+G: Subset4, GTR+I+G: Subset5;

end;

begin mrbayes;

log start filename = log.txt;

outgroup Caliscelis wallengreni;

outgroup Cixius sp;

outgroup Dicranotropis hamata;

outgroup Dicranotropis europaea;

outgroup Trypetimorpha occidentalis;

charset Subset1 = 1–1941;

charset Subset2 = 1942–2732;

charset Subset3 = 2733–3576;

charset Subset4 = 3577–4302;

charset Subset5 = 4303–4929;

partition PartitionFinder = 5: Subset1, Subset2, Subset3, Subset4, Subset5;

set partition = PartitionFinder;

lset applyto = (1) nst = 6 rates = invgamma;

lset applyto = (2) nst = 6 rates = invgamma;

lset applyto = (3) nst = 6 rates = invgamma;

lset applyto = (4) nst = 6 rates = invgamma;

lset applyto = (5) nst = 6 rates = invgamma;

prset applyto = (all) ratepr = variable revmatpr = dirichlet (1, 1, 1, 1, 1, 1) statefreqpr = dirichlet (1, 1, 1, 1);

unlink statefreq = (all) revmat = (all) shape = (all);

mcmcp ngen = 30000000 nruns = 2 relburnin = yes burninfrac = 0.25 printfreq = 1000 samplefreq = 1000 nchains = 4 savebrlens = yes;

mcmc;

sumt;;

end;
